# Salt Poisoning Due to Inadequate Infant Formula Preparation: A Rare Cause of Hypernatremia and Massive Cerebral Hemorrhage in a Newborn

**DOI:** 10.7759/cureus.33045

**Published:** 2022-12-28

**Authors:** Estela Kakoo Brioso, Joana Moscoso, Filipa Vieira, Anabela Salazar, Madalena Tuna

**Affiliations:** 1 Pediatrics, Centro Hospitalar Lisboa Ocidental, Lisbon, PRT; 2 Pediatrics, Hospital De Cascais Dr. José De Almeida, Lisbon, PRT; 3 Comprehensive Health Research Center, Nova Medical School, Lisbon, PRT

**Keywords:** infant formula, intracerebral hemorrhage, status epilepticus, salt poisoning, hypernatremia

## Abstract

Salt poisoning is a rare cause of severe hypernatremia in children resulting from the ingestion of toxic amounts of sodium chloride, either from accidental or intentional administration of salted solutions.

We present the case of a newborn admitted to a pediatric emergency department for lethargy and reduced oral intake; his laboratory evaluation showed severe hypernatremia ([Na^+^] of 174 mmol/L). The infant developed convulsive status epilepticus during treatment. Neuroimaging showed a tetraventricular hemorrhage, a large right-sided parenchymal hemorrhage with midline shift, and several left hemorrhagic foci. Etiologic evaluation for hypernatremia did not reveal a renal or extrarenal source of water loss nor an intercurrent illness to explain the reduced oral intake. A careful review of how the parents prepared the infant formula revealed an error in dosing the ratio of powder/water, resulting in hyperosmolar infant formula. The infant was diagnosed with salt poisoning as the major cause of hypernatremia. After careful correction of hypernatremia and the use of antiseizure medication, the patient improved and was discharged. The parents were given a careful review of instructions for infant formula preparation.

Due to its rarity, a high index of suspicion is mandatory for a correct diagnosis of salt poisoning. Timely and adequate treatment is needed due to the high risk of intracerebral bleeding, seizures, and irreversible neurologic injury. Children, particularly newborns and infants, depend upon adults to ingest water and, thus, have more difficulty in maintaining electrolyte balance. Therefore, it is of utmost importance that parents are educated about childcare, particularly on the importance of careful infant formula preparation.

## Introduction

Salt poisoning results from intentional or non-intentional ingestion of toxic amounts of salted solutions [1]. It is characterized by severe hypernatremia and neurologic symptoms resulting from cerebral cell shrinkage and intracranial hemorrhage [2]. Pediatric patients, and particularly infants, are especially prone to severe hypernatremia following ingestion of high quantities of sodium chloride since they cannot adequately express thirst and depend on adults to increase their water intake [3]. Most reports of salt poisoning are due to intentional administration in the context of child abuse or Munchausen syndrome by proxy but reports on unintentional administration also exist [1]. Inadvertent salt poisoning is the result of the use of salt as a folk medicine, due to its emetic and laxative proprieties, or inadequate infant formula preparation (either due to errors in powder dosage or the use of salted water) [1,4,5].

Children usually present to the emergency department with irritability, lethargy, or drowsiness, but seizures have also been described [5-7]. Laboratory evaluation shows a markedly elevated sodium concentration (often > 170 mmol/L) [1-3]. Kidney function might be normal or slightly impaired and urine osmolality is elevated, as well as the fractional excretion of sodium (> 2%) [3]. Differential diagnosis with the far more common hypernatremic dehydration is often difficult. Complications from salt poisoning are the result of high plasma osmolality and include intracranial bleeding, coma, irreversible neurological injury, and death [2]. Treatment of salt poisoning includes decontamination (in cases of acute ingestion) and correction of plasma sodium concentration [1]. The optimal rate of correction of hypernatremia in salt poisoning is unknown due to the rarity of this entity and the lack of observational and experimental studies [1]. Published evidence consists mainly of case reports and case series. In the lack of specific treatment guidelines, most articles report treatment according to established evidence for other conditions: acute hypernatremia (<48 hours) is treated with rapid repositioning of water deficit and dialysis when needed; chronic hypernatremia (>48 hours) is treated with hypotonic solutions aiming for correction rates of 0.5 mmol/L/h [1,2].

## Case presentation

We present the case of a male infant who was admitted to our emergency department. He was born at 36 weeks and two days of gestational age, as the second son of a nuclear family. Pregnancy surveillance was incomplete, with fewer than recommended office visits, but all three obstetric ultrasounds were normal. The third-trimester serology work-up was unremarkable, and the pregnancy was uneventful.

The peripartum period was also uneventful. He had a eutocic delivery, with no need for resuscitation in the delivery room (Apgar 10/10). Birth weight was 2815 g (50th percentile), length was 45 cm (10th-50th percentile), and head circumference was 33 cm (50th percentile). No feeding difficulties were reported in the post-natal period; micturition and defecation were normal in terms of perceived volume and appearance. He was discharged from the nursery on the fourth day of life, and exclusively fed on infant formula per the mother’s decision. At discharge, the mother seemed to have appropriate parenting skills, as well as reading ability.

He presented to the pediatric emergency department on his 14th day of life due to lethargy, reduced oral intake, and weight loss (-17.2% of birth weight). According to the mother, the infant maintained normal oral intake (around 440 mL of formula daily) until two days before admission, when the feeding difficulties started. Upon admission, the infant presented signs of dehydration (emaciation, diminished skin turgor, dry mucous membranes, and decreased tearing) but cardiovascular instability was not observed. No history of fever, vomiting, diarrhea or respiratory symptoms was encountered. The early diagnostic work-up showed severe hypernatremia ([Na+] 174 mmol/L) (Table 1). Venous blood gas analysis showed a pH of 7.12, PCO2 of 39 mmHg, HCO3- of 11.9 mmol/L, base excess of -15.5 mmol/L, and lactate concentration of 3.3 mmol/L. 

**Table 1:** Serial laboratory test results

Clinical parameters	14^th^ day of life (admission)	15^th^ day of life	16^th^ day of life	17^th^ day of life	18^th^ day of life
Hemoglobin (g/dL)	16.9	14.1	12.3	13.3	13.1
Leukocytes (x10^9^/L)	19.0	16.8	11.6	12.9	12.3
Neutrophils (%)	38	22	32	37	30
Lymphocytes (%)	48	67	57	45	49
C- Reactive Protein (mg/dL)	0.10	0.10	<0.10	<0.10	0.32
Urea (mg/dL)	132	92	18	10	10
Creatinine (mg/dL)	0.85	0.62	0.33	0.27	0.24
Sodium (mmol/L)	174	163	153	146	144
Potassium (mmol/L)	5	3.86	3.12	4.04	5.05
Chloride (mmol/L)	146	139	123	115	111

The infant was admitted and started on sodium correction with glucose 5% + NaCl 0.9%, aiming for correction of a water deficit of 394 mL over 58 hours with a maximum correction of [Na+] of 0.5 mmol/L/h. Despite the absence of elevated inflammatory markers and pending further investigation, the patient was started on intravenous cefotaxime and ampicillin until the exclusion of late-onset neonatal sepsis.

**Figure 1:**
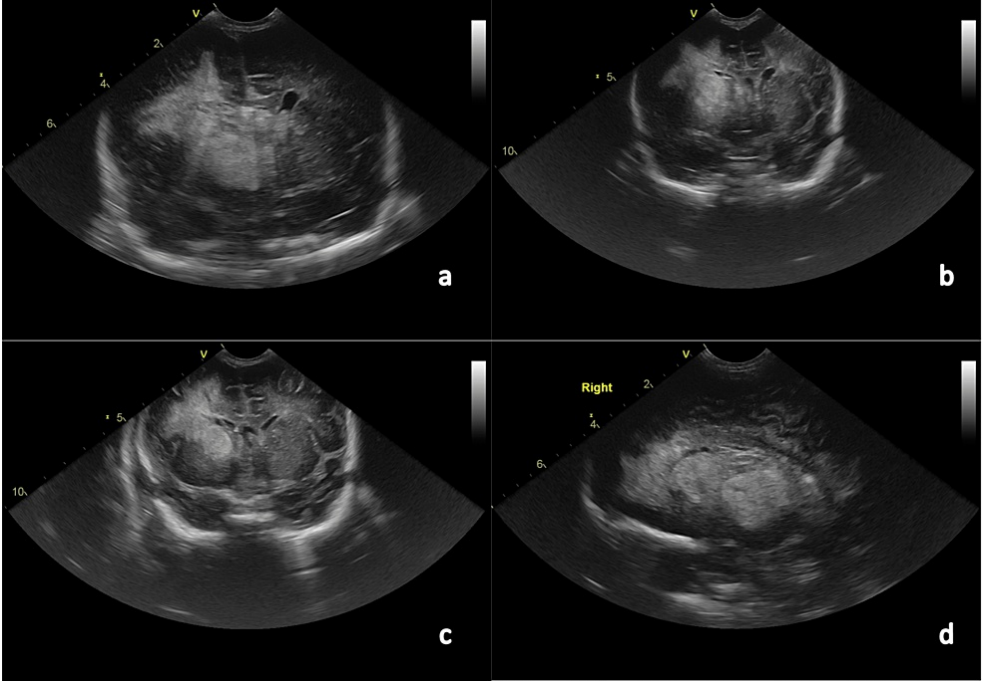
Cranial ultrasound showing tetraventricular hemorrhage with extensive right parenchymal hemorrhage, midline shift, and severe left fronto-parieto-occipital hemorrhage A,B,C: coronal views. D: parasagittal view.

**Figure 2:**
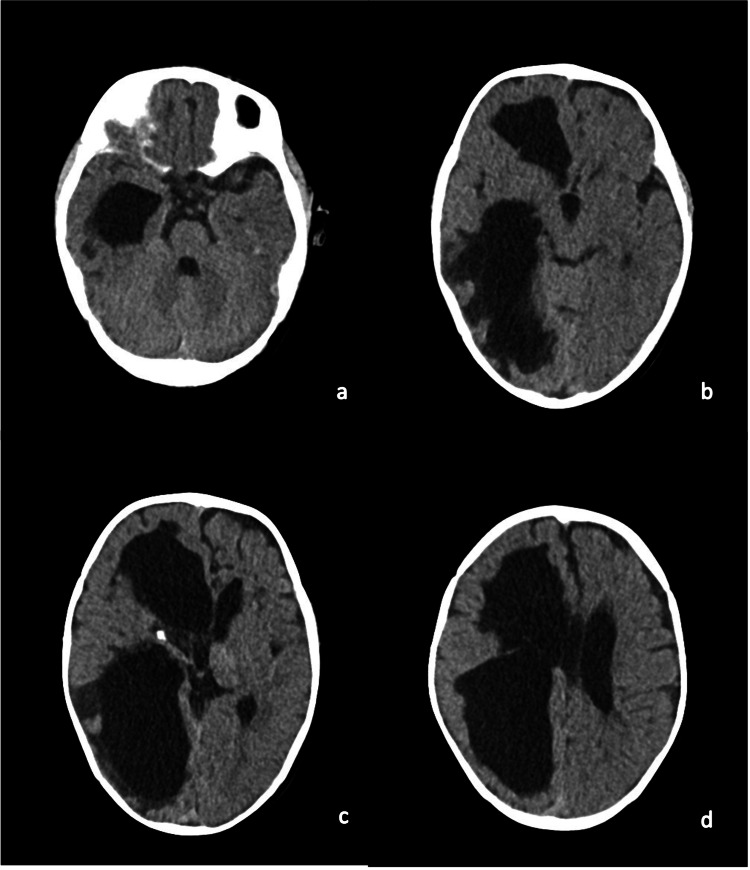
Head computer tomography showing intraventricular hemorrhage, hydrocephalus, brain edema, and right cerebral and left frontal hemispheric ischemia. A-D: axial views at different progressively superior planes.

At 36 hours post-admission, the infant progressed to a state of agitation, hypertonia with the extension of the upper limbs, and oromandibular movement. Acyclovir was added to the antimicrobial regimen. A cranial ultrasound was performed revealing a tetraventricular hemorrhage and a large right-sided parenchymal hemorrhage with midline shift, as well as several left frontal, parietal, and occipital hemorrhagic foci (Figure 1). Upon this finding, a diagnosis of convulsive status epilepticus secondary to intracranial bleeding was considered. The patient was transferred to the neonatal intensive care unit (NICU) and was monitored with amplitude-integrated electroencephalography (aEEG); he was administered phenobarbital (total of 40 mg/kg). Due to the maintenance of seizure activity, phenytoin (20 mg/kg) was administered but, ultimately, the infant needed to start midazolam infusion (0.06 mg/kg/h) and was put on invasive mechanical ventilation (IMV). A head CT scan was performed and showed similar findings to the ultrasound - massive tetraventricular hemorrhage with hydrocephalus, possible hypodense ischemic right-sided and left frontal lesions, and brain edema (Figure 2).

Sodium correction was maintained at the desired rhythm (with transition to a glucose 5% + NaCl 0.45% infusion when [Na+] dropped below 170 mmol/) throughout the course of the treatment of the status epilepticus and normal sodium was reached at 18 days of life.

Despite these measures, seizure activity was still present in the aEEG monitoring and levetiracetam (40 mg/kg, every eight hours) was administered, resolving the status epilepticus by the 21st day of life. The patient was weaned off the midazolam perfusion and the IMV and was extubated on the 23rd day of life.

Follow-up diagnostic evaluation showed no signs of sepsis or central nervous system (CNS) infection. Blood, urine, stool, and cerebrospinal fluid cultures were all negative, as were respiratory viral studies and polymerase chain reaction and serologies for herpes simplex virus. Etiologic evaluation of hypernatremia showed no evidence of renal water loss - urine osmolality was higher than plasma osmolality, excluding diabetes insipidus as a cause for hypernatremia. Additionally, no evidence of diabetes mellitus was found. We also could not find an extrarenal source of water loss. The absence of signs of infection or intercurrent illness to explain a diminished oral intake led to our conclusion that hypernatremia was probably the cause of the diminished oral intake present at admission rather than its consequence. Accordingly, a search for a source of salt poisoning was conducted. 

A careful review of the infant formula used at home revealed that the parents were preparing a preterm infant formula using four cup measures of formula for 60 mL of water ([Na+] = 60.6 mmol/L) (instead of the recommended one cup measure of formula for 30 mL of water - [Na+] = 30.3 mmol/L), resulting in a hyperosmolar infant formula. Consequently, the newborn was ingesting a total of 26.6 mmol of Na+ daily (9.5 mmol/kg/day), which is significantly above the recommended daily intake (2-5 mmol/kg/day for preterm newborns and 2-3 mmol/kg/day for term newborns). As such, we made the diagnoses of brain hemorrhage and convulsive status epilepticus secondary to severe hypernatremia due to salt poisoning from incorrect preparation of infant formula.

The infant was discharged from the NICU on the 45th day of life on oral levetiracetam treatment with referrals to pediatrics and neuropediatric appointments. The parents were educated on the proper instructions for preparing infant formula, as well as other important childcare themes.

## Discussion

Hypernatremia is a commonly encountered diagnosis in the pediatric emergency department. The most frequent etiology is hypernatremic dehydration, usually secondary to gastrointestinal water loss through diarrhea or vomiting or reduced water intake due to an intercurrent illness, as is an infection [8,9]. In newborns, the most common cause of hypernatremia is reduced intake due to inadequate breastfeeding [9]. Albeit less frequent, renal water loss is also an important mechanism of hypernatremic dehydration and it can sometimes be the first presentation of diabetes mellitus or diabetes insipidus [8].

Salt poisoning is a rare cause of hypernatremia and a high index of suspicion is mandatory for a correct diagnosis [1]. Although not usually associated with dehydration and weight loss, in small children unable to access water, the associated neurological symptoms, like lethargy and drowsiness, might lead to an accompanying reduced oral intake and concurrent dehydration, making this diagnosis even more difficult [3].

In our patient, the clinical and laboratory signs of dehydration and the reduced oral intake pointed to hypernatremic dehydration but a careful review of how the parents prepared the infant formula led to the suspicion of salt poisoning as the main cause of hypernatremia. The comprehensive investigation allowed the exclusion of other causes of hypernatremia. The presence of intracerebral hemorrhage was also more suggestive of salt poisoning as it is more frequently associated with this condition than with hypernatremic dehydration [1]. Although not well known, the more acute rise in serum sodium with salt poisoning and the consequent less time for cerebral adaptation to the hyperosmolar plasma seems to lead to increased cell shrinkage and vascular instability resulting in intracerebral hemorrhage [1,2]. Other causes of intracerebral hemorrhage in the newborn always need to be considered but they are most often encountered in the immediate postpartum period. Most frequently, intracerebral bleeding is secondary to the hemorrhagic conversion of an ischemic infarction [10]. Primary intracerebral bleeding in the newborn is most often idiopathic but sometimes can be related to hematologic conditions (e.g., thrombocytopenia or coagulopathies) or brain vessel anomalies (e.g., aneurism, cavernous malformations, or arteriovenous malformations) [10].

Intracerebral hemorrhage is an important cause of neonatal seizures and the most probable etiology for the status epilepticus of our patient [11,12]. Hypernatremia might also have contributed to seizure activity but hypernatremia-induced seizures are more often observed following acute shifts of natremia. Neonatal seizures are most frequently acute provoked seizures in relation to a specific etiology [11,12]. The most common etiology is neonatal encephalopathy but this is almost always related to a peripartum hypoxic event and usually presents in the immediate postpartum period. Acquired structural brain lesions (either ischemic or hemorrhagic), metabolic disturbances (e.g., hypoglycemia, hypocalcemia, and hypomagnesemia), CNS infections, or inborn errors of metabolism are also diagnoses to consider when evaluating seizures in a newborn [11,12].

In a patient with hypernatremia, a rapid decrease of natremia with treatment might also contribute to seizure, as well as brain edema and intracranial hypertension [2]. As such, careful monitoring of serum sodium levels during correction is fundamental in preventing iatrogenic complications.

## Conclusions

Salt poisoning is a rare hypernatremia etiology associated with an important risk of intracerebral bleeding, seizures, and irreversible neurologic injury. However, if diagnosed and treated promptly, the prognosis might be favorable. Children, particularly newborns and infants, depend upon adults to ingest water and, thus, have more difficulty in maintaining electrolyte balance. Therefore, it is of utmost importance that parents are educated regarding childcare, particularly on the importance of careful infant formula preparation.
